# Partnerships for safe care: A meta‐narrative of the experience for the parent of a child with Intellectual Disability in hospital

**DOI:** 10.1111/hex.12968

**Published:** 2019-09-27

**Authors:** Laurel Mimmo, Susan Woolfenden, Joanne Travaglia, Reema Harrison

**Affiliations:** ^1^ Faculty of Medicine School of Public Health and Community Medicine University of New South Wales Sydney New South Wales Australia; ^2^ Clinical Governance Unit Sydney Children's Hospitals Network Sydney New South Wales Australia; ^3^ Community Child Health Sydney Children's Hospital Sydney New South Wales Australia; ^4^ Faculty of Medicine School of Women's and Children's Health University of New South Wales Sydney New South Wales Australia; ^5^ Faculty of Health Centre for Health Services Management University of Technology Sydney Sydney New South Wales Australia

**Keywords:** child health, healthcare quality, hospitalization, Intellectual disability, patient experience, patient safety

## Abstract

**Objective:**

To systematically identify and synthesize peer‐reviewed qualitative evidence of the parental experience of hospitalization with a child with Intellectual Disability.

**Search strategy:**

Key words, synonyms and MeSH subject headings that related to the three key concepts of parental experience, children with Intellectual Disability and hospital settings were applied to six electronic databases: Medline, CINAHL, Embase, PsycINFO, Scopus and Web of Science. Titles and abstracts of publications between January 2000 and February 2019 were screened for relevance.

**Inclusion criteria:**

Empirical qualitative research involved participants aged 0‐18 years, involved children with Intellectual Disability, involved participants hospitalized as an in‐patient and involved participants focused on parent perspective.

**Data extraction and synthesis:**

Data were extracted and synthesized using a meta‐narrative approach.

**Results:**

Eleven publications met the inclusion criteria. Data synthesis revealed three research traditions contributing to this meta‐narrative: Paediatric Nursing Practice, Intellectual Disability Healthcare and Patient Experience. A total of five themes were identified: (a) being more than a parent, (b) importance of role negotiation, (c) building trust and relationships, (d) the cumulative effect of previous experiences of hospitalization and (e) knowing the child as an individual.

**Discussion and conclusion:**

This review presents a working model for professional‐parent partnership for the safe care of children with Intellectual Disability in hospital. Shifting paediatric healthcare to whole of hospital/multidisciplinary models of care that centre on the child will necessitate partnerships with the parent to identify and manage the needs of the child with Intellectual Disability, in order to achieve safe and equitable care for these children.

## 1 INTRODUCTION

Equitable, high‐quality and safe healthcare is the aspiration of healthcare systems globally to in order to achieve optimal patient outcomes; yet, despite concerted efforts over the past two decades, rates of avoidable harm have remained largely unchanged.^1^ Knowledge of patient experiences is increasingly recognized as critical to inform health systems regarding avoidable harm in healthcare delivery.^2^ Children are especially vulnerable to avoidable harms during hospitalization, predominantly those resulting from communication and medication errors.^3^ Yet, data regarding patient experiences amongst paediatric populations are challenging to collect and are often interconnected with parental or carer experience.^4^, ^5^ In the paediatric healthcare context, parents and carers are routinely used as proxies to obtain patient experience data.^5^, ^6^, ^7^

Certain paediatric populations have high healthcare utilization and may be exposed to increased risk.^8^ Children with Intellectual Disability (ID) are one such population,^9^ with emerging evidence which indicates that children with ID are particularly susceptible to avoidable harm in their care.^10^ Vulnerability to avoidable healthcare harm compounds the existing health inequities experienced by these children.^11^

Hospital staff rely on the presence of parents and carers to attend to the needs of children with ID.^10^, ^12^, ^13^ Being frequent users of healthcare, these parents or carers are therefore often more familiar with the health system and services than the general paediatric population. As such, parents of children with ID are uniquely positioned both in the role that they undertake and to report their observations of hospitalization. Reliable methods to collate patient experience data from children with ID are sparse except through proxy measures such as parents. Thus, parents are a valuable source of healthcare experience data for enhancing the experience of healthcare for children with ID and their parents or carers.^14^, ^15^

Parental experience of caring for a child with ID has been explored through the lens of several health disciplines, but exploration of parental experience from the quality and safety perspective is missing. This review aimed to identify evidence to date of the parental experience of hospitalization with a child with ID with regard to care quality and safety, and provide a consolidated narrative evidence synthesis.

## 2 METHODS

An initial scoping review of the literature identified a small number of key studies from a diverse range of research traditions with comparable findings, which would be ideally synthesized using a meta‐narrative approach. The meta‐narrative uses an iterative approach to the search strategy and aims to tell a story of the evolution of research into a specific tradition and its disciplines over time.^16^ The Realist and Meta‐narrative Evidence Syntheses: Evolving Standards (RAMSES) study standards^17^ were employed for conducting and reporting this review.

### 2.1 Eligibility criteria

#### 2.1.1 Inclusion criteria

##### Types of studies

Studies available in English and published since 2000 were eligible for inclusion. The year 2000 is contemporaneous with the study of two seminal healthcare Q&S texts, 18, 19 which stimulated growth in the study of patient experience from the healthcare quality and safety perspective.

##### Participants

Parents or carers of children (<18 years of age,aligning with the United Nations definition of child.^20^) with ID in hospital as inpatients. This could include either a specific condition known to include ID, such as Down syndrome, or terms that are synonymous with ID such as cognitive impairment, learning disability or developmental disability.^21^

##### Study design

Qualitative study designs and data are used to understand complex phenomena involving human interactions such as experiences of healthcare delivery, meaning they are ideal for capturing data on healthcare experience.^22^, ^23^

##### Outcomes

Parent/carer‐reported experiences of hospitalization, or any other terms referring to subjective measures of inpatient healthcare. The parental experience in hospital with a child with ID could be described using any of the following terms: satisfaction, experience or reporting quality of care.

#### 2.1.2 Exclusion criteria

Studies focussed on children with Autism only were excluded where the participants did not also have ID.^24^ Studies of short stay contexts were excluded as these present other concerns of the healthcare experience that have been explored elsewhere.^1^ Inpatient mental health contexts present unique challenges for children with ID, and their parents, including dual diagnosis^25^, ^26^ warranting separate study.

### 2.2 Study identification

**Figure 1:**
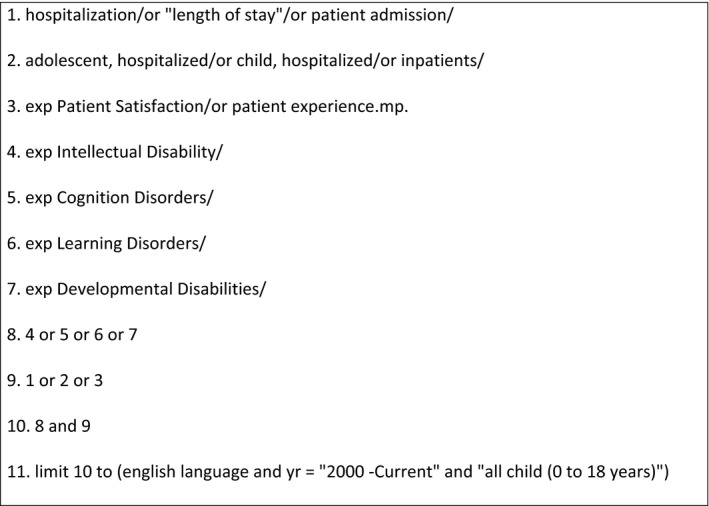
Example of search strategy in MEDLINE

A range of text words, synonyms and subject headings relating to patient experience, hospitalization, children and adolescents, and Intellectual Disability were used to systematically search six electronic databases from January 2000 to August 2019. Electronic searches were conducted from January 7 to January 13 2019, and February 18 to February 25 2019. The databases searched were as follows: MEDLINE, EMBASE, CINAHL, PsychInfo, Scopus, Web of Science and the Cochrane Library. An initial search was conducted in Medline, see Figure 1. Examples of the Boolean search terms applied are as follows: Intellectual Disability OR cognitive disorders OR learning disorders OR developmental disability; hospital*; experience OR satisfaction; infant OR child* OR adolescen* OR teenage*. The terms applied are synonymous with those used in other countries.^21^ Terms were adapted as necessary for subsequent searches in all other databases. Hand searching of reference lists of included studies and relevant journals, including *Learning Disability Practice, Journal of Intellectual Disabilities, Journal of Intellectual Disability Research, Journal of Applied Research in Intellectual Disabilities* and *Journal of Child Health Care*, was also used for completeness. Reference management software (Endnote ×9) was used to combine the results. Duplicates were removed.

### 2.3 Study selection and data extraction

Title and abstract screening was conducted by the author, and a copy of the full text was obtained for those studies potentially eligible for inclusion. Inclusion criteria were applied to these studies and discrepancies resolved through discussion with research supervisor. Data extracted included author(s), study year, country, health service setting, participants, study design, main focus and key findings.

### 2.4 Assessment of study quality

The Critical Appraisal Skills Programme (CASP) qualitative research checklist was used for the assessment of study quality.^27^ Each study was assessed for qualitative rigour against the ten CASP criteria and scored on a 3‐point, 0‐2 scale (No—0, can't tell—1, Yes—2) for a maximum score of 20 indicating a very high‐quality study. One author (LM) assessed all the studies, and uncertainties were resolved through discussion with another author (RH).

### 2.5 Data synthesis

Initial scoping searches of key electronic databases found small pockets of research in this area scattered amongst the broader research fields of nursing practice, disability healthcare and patient experience. With a small yet heterogeneous group of studies emerging, it was determined that data synthesis using a meta‐narrative was the best approach. A meta‐narrative is suited for sense‐making of phenomena as studied through different research perspectives and is presented as an evolutionary story of the topic.^16^

Each of the six phases of a meta‐narrative (planning, search, mapping, appraisal, synthesis and recommendations) is guided by six principles: pragmatism, pluralism, historicity, contestation, reflexivity and peer review.^16^ Applying these principles during each phase, the included studies were reviewed and research traditions and academic disciplines identified by one reviewer (LM). Summaries of how each research tradition was conceptualized across the included studies were completed. Each study was appraised individually before framing the data through narrative synthesis.

## 3 RESULTS

**Figure 2:**
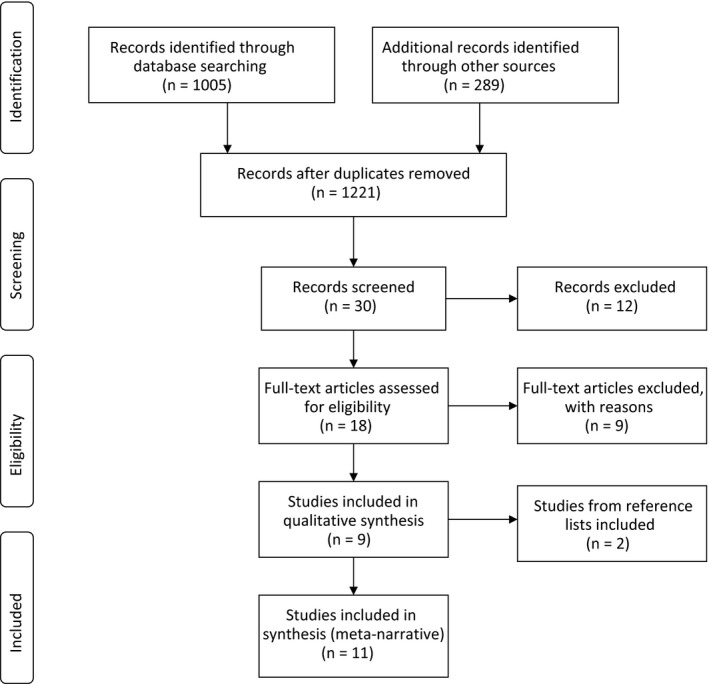
PRISMA 2009 Flow Diagram for study selection process^28^

A total of 1005 titles were identified from database searches, 932 after duplicates removed. From relevant journal searches, 289 titles were identified. After title screening, 22 were retained from the database searches and eight retained from journal searches for abstract or full‐text review. Of these, five studies from the database search and four studies from journal searches were included. Hand searches of the reference lists of these studies identified a further two studies for inclusion. A total of eleven studies were included in this review (see Figure 2 for PRISMA flow diagram of study selection^28^).

### 3.1 Characteristics of included studies

A summary of the studies included in this review is provided in Table 1. The 11 included studies reported findings from 10 unique data sets. Three studies were from the United Kingdom, two each from Canada and Sweden and one from each of the following: Australia, Norway, Switzerland and the United States. Two studies each discussed themes derived from a single data set.^29^, ^30^ Two studies used mixed methods^15^, ^31^ with only findings from qualitative analysis included in this review.

**Table 1:** List of included studies in meta‐narrative with quality assessment scores

Author(s)	Year	Journal	Study location	Setting	Discipline	Study design	Study population	Children's ID diagnoses (where specified)	Aims of the study	Methodology	Findings/themes	CASP score^27^
Aston, M., Breau, L., & MacLeod, E. 29	2014 (a)	Journal of Intellectual Disabilities	Canada	Single centre	Nursing	In‐depth interview	17 mothers, 12 nurses and 8 children	Autism spectrum disorder, developmental delay/Intellectual Disability, global developmental delay, chromosome disorder, cerebral palsy or other	‘The purpose of the present study was to better understand the personal, social, and institutional hospital experiences of children with IDs, their parents, and the nurses who cared for them.’ (p223)	Feminist poststructuralism; discourse analysis	Themes within Building relationships: The personal valuing of relationships; The institutional valuing of relationships, with; a) the role of time in relationship development; b) communication in relationship development; c) fear in relationship development; and d) when relationships work!	14
Aston, M., Breau, L., & MacLeod, E. 30	2014 (b)	Journal of Intellectual Disabilities	Canada	Single centre	Nursing	In‐depth interview	17 mothers, 12 nurses and 8 children	ID diagnoses included autism, foetal alcohol syndrome and global developmental delay	‘The purpose of the study was to better understand how children with IDs, their parents and nurses experience care whilst interacting with each other during the child's hospital visits.’ (p293)	Feminist poststructuralism; discourse analysis	Themes within diagnoses, labels and stereotypes: Diagnoses and labels help negotiate care; When labels shift to stereotyping; Challenging stereotypes; Children with IDs labelled as unable to communicate and understand; Children with IDs labelled as difficult patients; Parents of children with IDs labelled as difficult or bad parents;	16
Avis, M and Reardon, R. 33	2008	Journal of Child Health Care	United Kingdom	Single centre	Nursing	Purposeful sampling; Semi‐structured interviews	12 parents of children with learning disabilities and complex health needs	Not specified	‘…exploring parents’ views of the nursing care that their child with additional needs had received in hospital.’ (p8)	Thematic analysis	Four themes: Prior experiences of hospital care; Communicating support; Nurse‐parent relationships; Parents' perceptions of nurses and nursing.	20
Brown, FJ, and Guvenir, J. 12	2008	British Journal of Learning Disabilities	United Kingdom	Single centre; general hospital ward	Psychology and Nursing	Semi‐structured interviews	13 carers of inpatient children with learning disabilities; 13 nursing staff from the admitting unit; 2 children with LD	Not specified	To describe ‘the experiences of children, their families and staff during a hospital stay.’ (p111)	Thematic analysis	Five themes: 1. Child, carer and staff anxiety; Preparedness for admission; Difficulties managing the child's behaviour; Carer presence during the admission; Ward environment and facilities.	11
Downs, J., Torode, I., Ellaway, C., Jacoby, P. Bunting, C., Wong, K., Christodoulou, J., & Leonard, H. 15	2016	Developmental neurorehabilitation	Australia; national database	National database	Physiotherapy, Medical, Biostatistician,	Longitudinal study (data EXCLUDED) and open‐ended questionnaire	Families of 392 patients in the Australian Rett Syndrome Database (ARSD). Satisfaction data collection based on median age at scoliosis surgery of 13 y 1 month (7 y 1 month – 17 y 11 months)	Rett syndrome	Qualitative data only: ‘…explored family explanations of satisfying or dissatisfying clinical care.’ (p 32)	Content analysis of qualitative data	Themes: Relationships with healthcare professionals; Care in the hospital; Longer term issues.	12
Graham, R. J., Pemstein, D. M., & Curley, M. A. 14	2009	Critical Care Medicine	USA;	Single centre, PICU	Medical, Social Work and Nursing	Exploratory, qualitative study using semi‐structured interviews	8 parents (7 mothers, 1 father) of children with severe antecedent disabilities	Chromosomal anomaly, multiple anomalies, tuberous sclerosis, spastic quadriplegia with severe mental retardation, epilepsy syndrome, spinal muscular atrophy type II, multiple congenital anomalies	'To describe the experience of paediatric intensive care hospitalization from the perspective of parents of children with severe, antecedent disability.' (abstract)	Qualitative analysis of data	Seven major themes: know my child's baseline; integrate and bridge multiple services; disconnect between role of parent at home versus parent in the PICU; a PICU admission does not equate with respite; high stakes learning environment; heterogeneity within group; and lack of fit within the acute care model.	18
Hagvall, M., Ehnfors, M., and Anderzén‐Carlsson, A. 34	2016	Journal of Child Health Care	Sweden	Single centre; admitted to a paediatric ward at a university hospital	Nursing	Semi‐structured interviews	7 mothers and 2 fathers of children with ID	Diagnoses included hydrocephalus, cerebral palsy, myelomeningocele, epilepsy or autism	‘to describe parental experiences of caring for their child with medical complexity during hospitalization for acute deterioration. The specific aim was to study parental needs and their experiences of the staff's attitude.’ (p69).	Inductive content analysis	A single theme: ‘a balancing act between acting as a caregiver and being in need of care, illustrating the vulnerable situation at the hospital, where the parents served as the child's ambassador in various ways.’ (p71). Two subthemes with several subheadings Being in a vulnerable parental situation; Acting as the child's ambassador.	18
Iversen, AS, Graue, M., & Råheim, M. 13	2013	International Journal of Qualitative Studies on Health and Well‐being	Norway	Single centre; surgical unit	Nursing and Physiotherapy	Purposeful sampling; In‐depth interview	Interviews with 9 parent groups (3 mothers only, 3 mothers & fathers, 3 fathers only) of 9 children	Cerebral Palsy, all with some degree of speech impairment and ID, and other co‐morbidities.	‘This study explored the lived experiences of parents of children with CP undergoing surgery, as they describe them.’ (p2)	Analysis grounded in hermeneutic phenomenology	Core theme: At the edge of vulnerability ‐ being parents at hospital of a child with Cerebral Palsy undergoing surgery. Three subthemes: Establishing trust; Awareness of a child who cannot speak; Sensing bodily reactions.	19
Seliner, B., Latal, B., & Spirig, R. 31	2016	Journal for Specialists in Pediatric Nursing	Switzerland	Single centre, six paediatric units	Nursing and Medical	Cross‐sectional study with qualitative questions	Qualitative data: 24 mothers, 2 fathers	For qualitative interviews not specified	‘Aimed to assess parental burden of care, satisfaction with family‐centered care, and quality of life (HRQoL) of parents and their hospitalized children with profound intellectual and multiple disabilities (PIMD), and determine the relationship among these factors.’ (p148)	Content analysis	Three main concepts: Concerns for the children's well‐being; Parents’ effort; Support needs	13
Sharkey, S., Lloyd, C., Tomlinson, R., Thomas, E., Martin, A., Logan, S., and Morris, C. 4	2016	Health Expectations	United Kingdom	Paediatric wards in two general district hospitals	Nursing and Medical	Convenience and purposeful sampling, semi‐structured interviews and focus groups.	12 mothers, 1 father, 2 both parents; 2 multidisciplinary focus groups; 4 nurse interviews	Disabled children with communication difficulties	‘To explore experiences of ward staff and families regarding communication with children with ‘communication difficulties’ while inpatients and to use the information to identify barriers and facilitators to effective communication.’ (p739)	Thematic analysis and The Framework Approach	Five key themes (and several subthemes) from parent interviews: Knowing the child; Prioritizing communication; Parent‐professional relationship; Not enough time; Child's eye view.	18
Thunberg, G., Buchholz, M., and Nilsson, S. 32	2016	Journal of Child Health Care	Sweden	University research centre	Speech Pathology, Occupational Therapy and Nursing	Focus group interviews	10 mothers divided into three focus groups (4, 4 and 2).	Described as communicative disabilities ‘varied greatly, from multiple disabilities with no speech and restricted understanding of verbal communication to a specific language impairment.’ (p226)	‘To investigate parents’ experiences of the hospital visits together with their children with communicative disabilities and to collect their ideas about how to optimize communication in this situation.’ (p225)	Retrospective qualitative content theory	Four theme categories and 17 subcategories. Four themes: The importance of communication and understanding between child and staff; The importance of knowledge and skills in augmentative and alternate communication and special needs; The need of individualized care; Perceived safety due to interaction and environment.	13

Of the 11 included studies, nine used semi‐structured or in‐depth interviews for qualitative data collection, one used parent focus group interviews, 32 and one used open‐ended questions embedded in a questionnaire.^15^ Methods for the analysis of interview data included discourse analysis,^29^, ^30^ thematic analysis,^4^, ^12^, ^33^ content analysis^15^, ^31^, ^32^, ^34^ and hermeneutic analysis.^13^ One study did not specify the analytic strategy.^14^ The studies identified were published in the last 10 years, with one study from 2008,^33^ two published in 2009^12^, ^14^ and the subsequent eight studies published from 2013 onwards.

Of the studies reviewed, eight recruited participants from a single site, one recruited from two sites,^4^ one identified participants through relevant user organizations,^32^ and one recruited from the Australian Rett Syndrome Database.^15^ Participant selection and recruitment was based on a recent admission to hospital with their child. All studies included parent/carers as study participants. A total of eight studies specified the participants as mothers, fathers or both; in three of these studies, participants were mothers only,^29^, ^30^, ^32^ three studies included either parent,^14^, ^31^, ^34^ and two studies included either or both parents.^4^, ^13^ Participants were primarily mothers, but four studies included data collected from children with ID and/or healthcare staff.^4^, ^12^, ^29^, ^30^ These data were not included in this review.

Diagnosis was included in only five studies^14^, ^15^, ^29^, ^30^, ^34^ and included conditions causing developmental delay, chromosome disorder or anomaly, cerebral palsy, autism, Rett syndrome, tuberous sclerosis, spastic quadriplegia with mental retardation, hydrocephalus, myelomeningocele, epilepsy, spinal muscular atrophy and multiple congenital anomalies. A further two studies described the participants' children as having communicative disabilities.^4^, ^32^

Quality of the included studies varied, ranging from moderate (11/20) to very high quality (20/20); six studies scored 16 or above (see Table 1). Discussion of researcher reflexivity was inconsistent across the studies, and responder validation was lacking. Due to the low number of studies identified, study quality was not an inclusion criterion.

### 3.2 Findings

A total of three research traditions contributed to this meta‐narrative: Paediatric Nursing Practice, Intellectual Disability Healthcare and Patient Experience. Though these traditions are different, each reflects the broader perspective and principles of family‐centred care (FCC). The research traditions and their conceptualizations of the hospital experience for parents of a child with ID are summarized in Table 2.

**Table 2:** Research traditions, academic disciplines, scope and key concepts

Research tradition	Academic discipline	Definition and scope	Conceptualization of hospital experience for parents/carers of child with ID	No. of studies
Paediatric nursing practice	Nursing	The study of health and healthcare delivery for children, aged 0‐18 years	Communication Relationships Parent perceptions of the role of the nurse Parent perceptions of family‐centred care	3
Intellectual (learning) Disability health	Multidisciplinary	The study of health and healthcare delivery for people, including adults and children, with Intellectual Disability	Information sharing and partnerships in care Person‐centred Supporting the needs of the person and family Access to and preparation for hospital	3
Patient experience	Multidisciplinary	The study of the patient experience of health and healthcare delivery	Perception of care delivery from the patient or parent lens	5

All studies were conducted by healthcare professionals with the nursing perspective leading or contributing to nine of the 11 studies. Of the studies reviewed, four were conducted from a nursing perspective only^29^, ^30^, ^33^, ^34^ with the remaining seven studies including researchers from other health disciplines such as medical, psychology, physiotherapy, speech pathology and occupational therapy.^4^, ^12^, ^13^, ^14^, ^15^, ^31^, ^32^

Through the review process, findings from the eleven studies were consolidated into five themes; being more than a parent, importance of role negotiation to reduce ambiguity about the role of the parent, building trust and relationships through effective communication, the cumulative effect of previous experiences of hospitalization, and healthcare staff (HCS) taking time to know the child as an individual. The themes crossover, they interrelate to tell the story of the parental experience over time, and how the interactions between the themes highlight the importance of partnerships in care to deliver safe care for children with ID. The five themes are detailed below:

#### 3.2.1 Being more than a parent

Parents consistently reported that HCS relied on their constant presence, assuming parents would take on multiple roles and provide the necessary care for the child with ID. Coupled with the stress of hospitalization, this impacted on the burden of responsibility for parents in several ways.

They relied on us like another member of staff. parent^12^(p113)

Parents reported they were expected to and relied on by HCS to monitor, protect, speak for and advocate for their child with ID,^4^, ^12^, ^13^, ^15^, ^29^, ^30^, ^31^, ^32^, ^33^, ^34^ or HCS left the parent alone to do everything and look after themselves^4^, ^13^, ^34^:

it felt like we were, we had our camp there and they would come in to do what they had to do with the medication but otherwise left us to it, and that made me uncomfortable as because I had a younger son I could not be there all the time. parent^4^(p744)

Some parents perceived that HCS reliance on parents meant their child was ignored^4^, ^30^, ^33^ or the parents were left to attend to aspects of their child's hospital care the parent considered to be the role of HCS.^12^, ^33^ Parents described the assumption of multiple roles and perceiving an expectation to take responsibility for their child's care, reported feeling overwhelmed by this perception of reliance and need to be omnipresent,^12^, ^13^, ^15^, ^31^, ^33^ vigilant in watching over their child^15^, ^34^ and protective, as one parent commented, “…*you are their bodyguard*.”^14^(p2067) In contrast, being considered experts in their child's care was valuable to parents,^14^, ^15^, ^30^, ^33^ and parents recognized the benefit of sharing expertise with HCS for mutual learning about their child.^13^, ^14^

Parents consistently reported the burden of responsibility for making decisions on behalf of their child; parents felt guilty for consenting for treatment that subjected their child to both pain and discomfort 13, 15, 31or that their decision could be wrong.^13^, ^14^, ^34^ One parent expressed torment about the legitimacy of their decision:

Did I take the right decision? parent^13^(p6)

Two studies identified this burden of responsibility and ensuing guilt created a tension for parents.^13^, ^15^ HCS reliance on parental presence created a sense of helplessness and vulnerability for the parent,^13^, ^31^, ^34^ or for their child.^31^ For some parents, the burden was overwhelming and contributed to feelings of guilt^15^, ^30^ and chronic sorrow.^33^

#### 3.2.2 Importance of role negotiation for shared care in the context of ambiguity

Parents expressed uncertainty and ambiguity about who was in charge of their child's care when they perceived that HCS did not provide sufficient information to enable them to negotiate care roles.^12^, ^13^, ^31^, ^33^, ^34^ Role ambiguity amongst parents in the absence of adequate role negotiations and partnerships with HCS contributed to parents feeling unable to leave their child's bedside due to safety concerns.^12^, ^13^, ^30^, ^33^, ^34^ This led to parents perceiving they could not trust that HCS had the capacity or knowledge to provide safe and high‐quality care to their child.^12^, ^13^, ^31^, ^33^, ^34^

We’re the only parents who are forced to view our child completely objectively. Otherwise you never need to do that as a parent […] parent^34^(p73)

Parents wanted HCS to recognize that the parent was in need of care and support as well.^12^, ^13^, ^14^, ^31^, ^33^, ^34^ In two studies, parents reported that the perceived expectation to be constantly available was a significant burden^13^, ^34^:

It is tough to be given the responsibility; we have to stay awake all the time. mother^13^(p6)

Parents reported HCS did not involve them in decision‐making or dismissed their expertise, yet were expected by HCS to be the expert for all aspects of their child's care.^14^, ^15^, ^29^, ^30^, ^31^, ^34^ Parents identified tensions with being in control of their child's care at home then losing autonomy while their child was hospitalized^14^, ^31^, ^34^:

I am always a little bit shocked when I come into the ICU. My medicines have to be inspected and I do not do anything. I mean, I try to help and sometimes I do and sometimes I am told well very kindly just to step aside, which I do. I do not argue with that but we are expected to be experts at home and we are not always experts here. In fact, most of the times we are not. parent^14^(p2066)

Tensions and the burden on parents can be reduced if HCS negotiated and clarified with parents about roles in their child's care needs,^13^, ^14^, ^30^, ^31^, ^33^, ^34^ minimizing the associated ambiguity.

#### 3.2.3 Building trust and relationships through effective communication

HCS reliance on parental presence contributed to role ambiguity, hindering opportunities for shared learning and negotiating care, and this created barriers to building trust and relationships with the child with ID and their parent. Parents reported the importance of HCS taking the time to build relationships with the parent and child.^4^, ^12^, ^13^, ^14^, ^15^, ^29^, ^31^, ^32^, ^33^, ^34^ Building relationships was important for understanding the support needs of the parent^13^, ^14^, ^29^, ^33^ and to promote parental trust in HCS^4^, ^33^, ^34^:

[…] It is too much for me to take that responsibility. I do not have enough knowledge; I can be wrong. father^13^(p6)

Sharing information, continuity of HCS caring for their child, and recognizing and respecting the expertise of the parent fostered trust.^13^, ^14^, ^29^, ^31^, ^34^ Parents reported feeling secure or confident with HCS who listened to them and respected their expertise^4^, ^12^, ^13^, ^14^, ^31^:

Health professionals have to listen to the parents when they are interpreting the child. For example, when hospitalization arouses feelings of anger and fear, they have to plan the intervention together with the parents and at least prepare both the parents and the child for what is going to happen. father^13^(p6)

Parents recognized that HCS may fear how to care for and communicate with children with ID^12^, ^29^ or may lack the necessary experience^4^, ^32^ and this was perceived as a barrier to HSC building relationships with the child.^29^ Parents valued HCS who communicated directly with their child or took time to create rapport noting this was often experienced HCS^4^, ^13^, ^29^, ^32^, ^33^:

he came and sat down next to C on the bed […] some informal chatting, where do you go to school? […] And so you get a rapport going with the child. parent^4^(p746)

#### 3.2.4 The cumulative effect of previous experiences of care during hospitalization

Parents consistently identified aspects of previous poor experiences of hospitalization and continuity of care that influenced their expectations of care for present and future hospitalizations. Memories of past hospitalizations inform, as one parent said, their ‘hospital career’:^33(p12)^.

Thankfully, it is not my first ICU visit so I anticipated it being completely horrific. I always plan for the worst.[…] parent^14^(2067)

Sharkey et al identified ‘previous negative experiences may have led parents to seem negative, defensive or combative’^4(p748)^ and this may contribute to parents of children with ID feeling stereotyped as difficult by HCS.^30^ Past experiences increased their anxiety about having negative experiences during the next admission,^12^, ^13^, ^14^ and in one study, this contributed to a sense of ‘chronic sorrow’ for these parents^33(p12)^.

Past experiences in which there was an apparent lack of continuity of care during a hospital admission also impacted on parental perceptions and expectations of care provided for their child during hospitalization. Parents reported feeling anxious about their child's safety during hospitalization when the nurse did not know their child,^12^, ^32^ or HCS were not prepared for their child's hospitalization.^12^ Parents reported that inconsistency of HCS led to poor pain management and a lack of information sharing during their child's hospitalization,^4^, ^13^, ^15^, ^32^ lessened their confidence in the HCS^15^ and impeded partnerships in care.^4^, ^14^

Conversely, continuity of HCS, especially those already known to the parent and/or child from previous hospitalizations, had a positive impact on the hospital experience^4^, ^12^, ^14^, ^15^, ^29^, ^32^, ^34^:

…The times we’ve come in and it’s been great that’s when we see nurses that we know, or with whom everything works well, and who understands the child. And when the doctor who knows the child best is on duty and everything works. parent^34^(p72)

Parents identified sharing of expertise and knowledge was important for trusting HCS with their child.^4^, ^14^, ^29^, ^30^, ^31^, ^34^ Being included in the care of their child during hospitalization was an opportunity for shared learning with HCS,^4^, ^14^, ^29^, ^30^, ^32^, ^34^ and for continuity in the care, they would provide at home.^14^

#### 3.2.5 Healthcare staff taking time to know the child as an individual

The importance of HCS and organizations recognizing the child with ID as an individual with unique needs during hospitalization was identified across most studies.^4^, ^12^, ^14^, ^29^, ^30^, ^32^, ^34^ When HCS did not take the time to get to know their child, the parent perceived their child was marginalized^29^, ^30^ or unnoticed by HCS.^4^ Parents gave tacit expressions of a need for HCS to see their child with ID as a human being, with a personality^4^, ^12^, ^13^, ^14^, ^29^, ^30^, ^31^, ^32^, ^33^, ^34^:

I try to bring in pictures of her, something to show that this is what she is really like. Because they do not know. They really do not…It is good for them to see a picture of what she is really doing and to realize that she is pretty interactive and understanding of stuff. parent^14^(p2066)

Treating a child with ID like any other child did not deliver the same quality of care for several reasons; because the child's needs did not fit with the acute care model,^14^, ^32^ it caused disruption^12^ or meant the child was ignored^4^:

I know that they are really difficult because they are really busy, but if B was, and I hate to say it, if B was a ‘normal 14 year old’ child then he would be demanding the Xbox or his food and wanting this and that and they would have to spend their time getting it for him […] He is not getting their time […] parent^4^(p743)

Parents reported HCS made negative assumptions about the child with ID’s cognitive ability, capacity to communicate or behaviour instead of making adaptions to accommodate their child.^4^, ^12^, ^14^, ^29^, ^30^, ^34^ Parents perceived they and their child were unimportant when HCS made negative assumptions about their child,^30^, ^34^ and reported feeling unsupported by HCS^4^, ^12^, ^34^:

There are actually quite a few nurses who said, in a somewhat irritated way, Oh my, you sure are getting tense, you’re going to have to relax now. And all I can think is, don’t you know anything about cerebral palsy? parent^34^(p71)

Many parents indicated it was important HCS know their child as a person; they appreciated HCS who communicated with their child^4^, ^30^, ^34^ who took the time to listen to them, hear their concerns and provide sympathy,^12^, ^13^ and spent time getting to know their child^14^, ^29^, ^33^ treating the child as a person.^34^ When HCS knew a child's unique needs, they could make adaptations to the hospital environment and optimize the care experience^12^, ^14^, ^32^:

now we mostly visit the emergency department…they have actually been very generous and offered an examination room if needed, otherwise it would be quite difficult in the waiting room. mother^32^(p231)

### 3.3 Partnerships in care

The value of partnerships between parents and HCS to help care for and make decisions about their child's care needs during hospitalization permeated each research tradition.^13^, ^14^, ^15^, ^29^, ^32^, ^34^ Parents wanted to work in partnership with HCS when making decisions about their child's care, as they cannot be objective.^13^, ^34^ Parents expressed value in partnerships with HCS to help care for and make decisions about their child's care needs during hospitalization.^13^, ^14^, ^15^, ^29^, ^34^ Two studies highlighted that parents viewed participation in the study as an opportunity to be heard, talk about their opinions and share their experiences of hospitalization with their child with ID.^13^, ^33^

**Figure 3:**
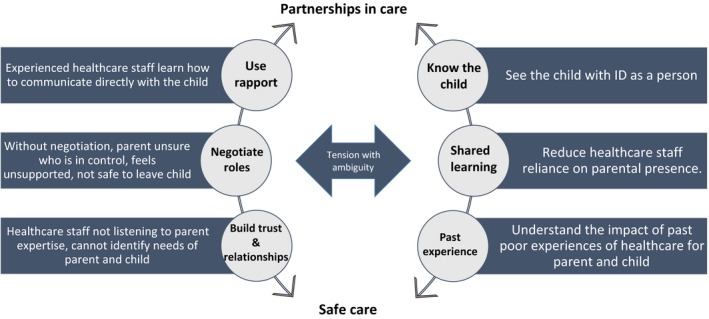
Conceptual model for safe care of a child with ID in hospital

Based on the findings of this review, we propose a conceptual model of how these five themes may interact in practice to support the development of partnerships between HCS and parents to deliver safe care for children with ID in hospital. This proposed conceptual model is presented below in Figure 3.

## 4 DISCUSSION

There were five themes elucidated across the eleven included studies, precursors for the development of partnerships in care. This review found that, for parents, HCS build trust and relationships with parents by getting to know their child, negotiating care roles and working in partnership with parents, resulting in safe care. Partnerships in care between parents and HCS enabled sharing of expertise, meaning the parent was not left to manage and be responsible for all their child's cares and medical decisions alone, and the parent felt able to safely leave their child in the care of HCS.

The five themes presented in this review are punctuated by notions of time; building trust and relationships take time, parents perceived HCS have limited time to care for the child with ID hence they rely on parents to save time, and it takes time to negotiate care in partnership with parents. Brown and Guvenir found some nurses saw reliance on parents as a time‐saving strategy, helping manage their workload.^12^ Taking time saves time; a 2015 systematic review found poor communication and lack of role negotiation between nurses and families resulted in repetition of information, wasting the time of staff and families.^35^ Furthermore, as with all children, the specific needs of children with ID change with time, as the child grows^36^ necessitating renegotiation of roles and partnerships as expectations and needs change.

Reliance on parents compounded their existing burden and contributed to an overwhelming responsibility of caring for a child with ID, and similar findings have been described by others.^35^, ^37^ The parental need for support, role negotiation and partnerships in care have been consistently reported in the paediatric healthcare literature across a variety of populations and settings.^7^, ^37^, ^38^, ^39^, ^40^ Espezel and Canam (2003) note that it may be that current healthcare environments do not facilitate the parent‐nurse rapport and subsequent relationship development that precedes a perception of a partnership.^41^

It is essential for healthcare staff to demonstrate empathy, compassion and kindness to engage children and their parents in true partnerships that recognize personhood.^39^ Where healthcare delivery is not person‐centred, the child is not viewed as an individual while receiving healthcare. This may lead to unnecessary suffering and dehumanization of the child.^42^ Furthermore, a recent review found that people with ID, irrespective of their degree of self‐awareness, do not consider their ID as a critical component of their self‐identity.^43^ Keeping the child and their individual needs at the centre of the care experience acknowledges the child has intrinsic value, a humanness and personhood, aligning with principles of person‐centred care.^44^

### 4.1 Implications

Models of paediatric healthcare that centre on the child and their healthcare needs will inherently include partnerships with parents, while maintaining focus on the personhood of the child. Such a change will necessitate a systems‐wide approach to improvements such as health policy^31^ and enhanced undergraduate education for healthcare professionals.^30^ Yet to shift values and beliefs around the personhood of the child with ID would necessitate broader social and health system changes.^30^

While governing health bodies around the world promote inclusion and reasonable adjustments for people with disabilities in hospital, HCS may lack the necessary understanding, capabilities and resources to implement changes.^45^, ^46^ With our conceptual model, we argue for achieving safe and equitable healthcare for children with ID is the goal, realized through partnerships in care and founded on HCS reducing the parental burden through role negotiation, using effective communication to build trust and relationships, recognition of previous poor experiences and getting to know the individual needs of the child with ID. This model will be tested in future qualitative studies.

### 4.2 Limitations

We have identified several limitations of this meta‐narrative, which may impact the generalizability of our findings. Firstly, the literature on this topic, while seeming to come from different research traditions, was inclined towards the paediatric nursing discipline. Most studies spoke to the parental experience with nursing staff, though for this review the term HCS encompasses any clinical disciplines providing acute care within the inpatient hospital setting. Patient experience studies of inpatient care will unavoidably overreport aspects of nursing care as nurses are the key contact for patients. However, this means these findings cannot be generalized to encompass the healthcare experience outside the inpatient setting.

Another limitation is that participants were chiefly the child's mother, meaning fathers and other family members or caregivers are underrepresented in the research. While an overrepresentation from mothers is to be expected, this has been previously identified by others as a potential bias.^40^ Recommendations to researchers include making conscious effort in study design and recruitment strategies to minimize this potential bias by using participant enrolment methods that are unlikely to favour mothers and may capture a broader range of perspectives from all carers involved.^40^

Where stated, the diagnoses of some participants, such as myelomeningocele and spinal muscular atrophy, were not specified to include ID. As this was a small number of children, the majority of children in each study had ID, and results were similar across the included studies to those of other parents, this is unlikely to have confounded the findings.

Finally, by limiting the included studies to English, some excluded non‐English studies identified during searches of the reference list may have been relevant. With a small number of studies included in this review, it is possible that this has impacted the generalizability of these findings in non‐English speaking settings and future studies would benefit from including this perspective.

### 4.3 Conclusion

This meta‐narrative describes a clear need for healthcare staff to develop partnerships in care with parents for there to be safe care for children with ID in hospitals. This starts by negotiating care and shared learning to lessen reliance on parental presence, building trust and relationships to identify the needs of the child with ID and their parent, understanding the impact of previous negative experiences of hospitalization and using rapport to get to know the child as a person. Models that centre on the child and their healthcare need to include negotiating care roles and partnerships with parents, while maintaining focus on the child. Shifting beliefs about the optimal models of paediatric healthcare will necessitate a systems‐wide approach to change the broader social and cultural perceptions of the value of people with ID.

We present these findings in a conceptual model for safe care of the child with ID in hospital through the development of partnerships in care between healthcare staff and parents.

## CONFLICT OF INTEREST

All authors declare they have no conflict of interest.

## Data Availability

Data sharing is not applicable to this article as no new data were created or analysed in this study.
